# Draft Genome Sequence of a Symbiotic Bacterium, *Rhizobium vignae* CCBAU 05176^T^

**DOI:** 10.1128/genomeA.00657-14

**Published:** 2014-07-31

**Authors:** Yang Liu, Zhiwei Yi, Runying Zeng

**Affiliations:** a State Key Laboratory Breeding Base of Marine Genetic Resource; Third Institute of Oceanography, SOA, Xiamen, China; b Collaborative Innovation Center of Deep Sea Biology, Hangzhou, China

## Abstract

The *Rhizobium vignae* strain CCBAU 05176^T^ was isolated from a root nodule of *Astragalus dahuricus* grown in Hebei Province, China. It grows on yeast mannitol agar (YMA) supplemented with 0 to 2% (wt/vol) NaCl. We report the annotated genome sequence of this strain in a 6.34-Mb scaffold.

## GENOME ANNOUNCEMENT

Rhizobia were first proposed by Frank (1) and are Gram-negative rod-shaped bacteria that mainly cause the formation of root nodules on legumes and fix nitrogen inside the nodules; they belong to the alpha and beta subclasses of *Proteobacteria*, and the names alpha- and beta-rhizobia are used to distinguish them from each other (2, 3). Alpha-rhizobia are common symbionts of most legume species, and the beta-rhizobia described so far have an afﬁnity toward the *Mimosa* genus (3). *Rhizobium vignae* strain CCBAU 05176^T^ belongs to the alpha-rhizobia and was isolated and identified by Ren et al. (4). Here, we represent the draft genome of CCBAU 05176^T^ for further study of its symbiotic capability and taxonomic classification.

The genome of *R. vignae* CCBAU 05176^T^ was sequenced using the Illumina/Solexa MiSeq technology at the Shanghai Majorbio Bio-pharm Technology Co., Ltd. (Shanghai, China). A library with a fragment length of 500 bp was constructed, and a total of 1,476 Mbp paired-end reads of 300 bp were generated. Approximately 1,047 Mbp of high-quality reads, which provided a 165-fold depth of coverage, were assembled with SOAP*denovo* version 1.05, resulting in a total of 84 contigs. Protein-coding sequences were predicted by Glimmer software version 3.0 (5) and annotated using BLAST searches of the nonredundant protein sequences from the NCBI, Swiss-Prot, TrEMBL, COG (6), and KEGG (7) databases. Ribosomal RNA genes were detected using RNAmmer software version 1.2 (8), and transfer RNA genes were detected using tRNAscan-SE (9). Genes of interest were manually evaluated.

The *R. vignae* CCBAU 05176^T^ genome consists of 6,343,049 bases, with a G+C content of 61.6%. There are 6,173 putative coding sequences, 43 tRNA genes, and 3 rRNA clusters. Gene clusters that participate in the synthesis of T1 polyketide-type secondary metabolites were detected by antiSMASH 2.0. Functional annotation of the genome sequence was automatically done using the RAST server (10). The draft genome has functionally categorized genes under 467 subsystems. One gene coding for nitrogen fixation protein, *fixG*, and one gene coding for nodulation protein, *nodT*, were found, but no *nodD* or *nifH* genes were detected in this genome. Genome analysis and comparison to other legume symbionts will contribute to a further understanding of legume-*Rhizobium* symbiosis. These genome data will represent a solid platform for further characterization and exploitation of the metabolic features linked to legume-*Rhizobium* symbiosis and secondary metabolites.

### Nucleotide sequence accession number.

This whole-genome shotgun project has been deposited at DDBJ/EMBL/GenBank under the accession no. JNNU00000000.
